# A Retrospective Study of Hospitalizations in the USA: Proportion of Hospitalizations With Non-Alcoholic Fatty Liver Disease in Non-Obese Population

**DOI:** 10.7759/cureus.17869

**Published:** 2021-09-10

**Authors:** Olubunmi Oladunjoye, Adeolu O Oladunjoye, Rashmi Dhital, DilliRam Poudel, Oreoluwa D Oladiran, Ibiyemi O Oke, Gabriel Areoye, Asad Jehangir, Oluwaseun Shogbesan

**Affiliations:** 1 Internal Medicine, Reading Hospital - Tower Health System, West Reading, USA; 2 Psychiatry, Baylor College of Medicine, Houston, USA; 3 Medical Critical Care, Boston Children's Hospital, Boston, USA; 4 Rheumatology, Indiana Regional Medical Center, Indiana, USA; 5 Cardiology, Reading Hospital - Tower Health System, West Reading, USA; 6 Gastroenterology and Hepatology, Medical College of Georgia at Augusta, Augusta, USA; 7 Gastroenterology, Reading Hospital - Tower Health System, West Reading, USA

**Keywords:** non-alcoholic fatty liver disease, non-alcoholic steatohepatitis, non-obese nafld, lean nafld, hospitalization

## Abstract

Background

Non-alcoholic fatty liver disease (NAFLD), one of the leading causes of end-stage liver disease, is known to be associated with obesity. However, only a few studies in the United States (US) have described non-obese NAFLD, most of which were on the outpatient population.

Aim

We aimed to investigate the proportion of hospitalizations in the US with a diagnosis code that included NAFLD in the non-obese population.

Methods

We analyzed adult discharges from the Nationwide Inpatient Sample with a diagnosis of NAFLD from January 2010 to December 2014. We created two groups: obese (overweight or obese) and non-obese (normal or underweight) groups. Basic demographic and clinical characteristics were compared using the chi-square test and Student’s t-test*.*

Results

A total of 194,787 hospitalizations with NAFLD were identified over the five-year period. It was observed that the prevalence of non-obese NAFLD hospitalizations increased yearly. Non-obese NAFLD hospitalizations had a higher mean age (57.5 vs 51.5 years, p < 0.0001) and a higher proportion of males (43.3% vs 36.1%, p < 0.0001) than obese NAFLD hospitalizations. With univariate analysis, non-obese NAFLD hospitalizations had lower odds of hypertension (OR 0.74, p < 0.0001), diabetes mellitus (OR 0.65, p < 0.0001). Non-obese hospitalizations had higher odds of cirrhosis (OR 1.30, p < 0.001) and decompensated cirrhosis (OR 1.30, p < 0.001) after adjusting for age, sex, race, diabetes mellitus, and dyslipidemia. Hospitalizations with non-obese NAFLD had higher odds of death (OR 1.49, p < 0.001) after adjusting for age, gender, race, co-morbidities, cirrhosis, and liver decompensation.

Conclusion

There is a continued rise in the proportion of non-obese NAFLD among hospitalizations in the US. Non-obese NAFLD hospitalizations were less likely to have hypertension and diabetes, but more likely to have decompensated liver disease. Further studies are needed to better characterize these patients to enable early detection, treatment, and reduction in complications of liver disease.

## Introduction

Non-alcoholic fatty liver disease (NAFLD) is the most common cause of chronic liver disease affecting more than 33% of adults globally [1] and one of the leading causes of end-stage liver disease and hepatocellular carcinoma [2, 3]. NAFLD is associated with increased mortality and morbidity especially when coexisting with cardiovascular disease, type 2 diabetes, and other chronic conditions [4].

While NAFLD is known to be associated with obesity, type 2 diabetes mellitus, and metabolic syndrome, there are increasing reports of NAFLD in patients with normal body mass index (BMI) [4, 5]. This group described as “non-obese” NAFLD may have contributing risk factors including insulin resistance, weight gain within normal weight limits, visceral obesity, high cholesterol and fructose intake, genetic risk factors such as TM6SF2 and patatin-like phospholipase domain-containing protein 3 (PNPLA3) polymorphism [3, 5].

There is no standard definition for non-obese NAFLD globally because the majority of studies in the Asian population describe non-obese NAFLD at <23 kg/m^2^ while most studies in the European and American population put the BMI cut off at <25 kg/m^2^ or <30 kg/m^2^ in some cases. The worldwide prevalence of non-obese NAFLD has been reported to vary from 3-30% of patients with NAFLD [5]. Prevalence of non-obese NAFLD (with BMI <23 kg/m^2^) has been reported to be 8-19% in Asians [3]. In the United States, a study describing non-obese NAFLD (BMI <25 kg/m^2^) from 1988-1994 using the national health and nutrition examination survey III reported 21% prevalence of non-obese NAFLD in the adult population [5].

While this group of non-obese NAFLD has been extensively described in Asia [5], to the best of our knowledge, only a few studies [6, 7] in the United States (US) have described non-obese NAFLD population which is increasingly becoming relevant among those with NAFLD. There is limited data on histology and morphologic characterization of non-obese NAFLD generally [5]. Therefore, we carried out this study to investigate the proportion of non-obese NAFLD focusing on hospitalizations in the United States. We hypothesized that the proportion and disease burden of non-obese NAFLD among hospitalized patients is increasing. This article was previously presented as a poster at the 2021 DDW annual meeting on May 22, 2021.

## Materials and methods

Study design and data sources

We analyzed all adult (18 years and older) discharges from January 1st, 2010 to December 31st, 2014 using the Nationwide Inpatient Sample (NIS) database. This database is a part of the Healthcare Cost and Utilization Project (HCUP) and sponsored by the Agency for Healthcare Research and Quality (AHRQ) [8, 9]. NIS is the largest of all-payer publicly available inpatient care database in the US made up of more than 4500 hospitals from more than 40 states in the US representing over 7 million annual hospital discharge records representing 20% of the hospitalized population. Since the database is de-identified and publicly available, no ethical clearance or Institutional Review Board approval was required.

Study population and characterization of variables

**Figure 1:**
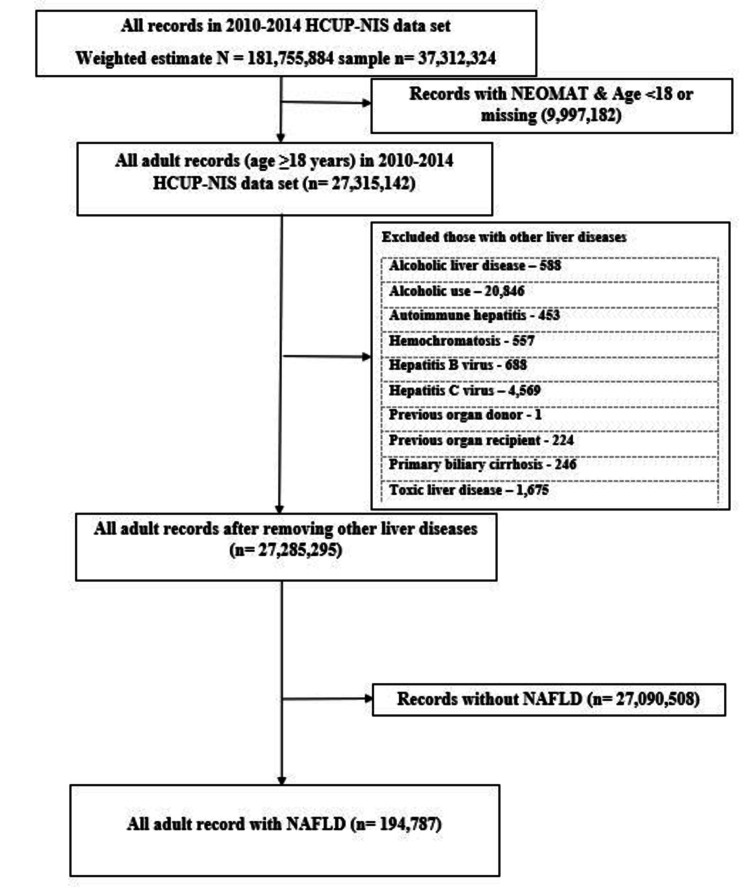
Flowchart for NAFLD US hospitalizations from 2010 to 2014 N: Weight average estimates; n: Sample size; %: Percentage; NAFLD: Non-alcoholic fatty liver disease; NIS: Nationwide Inpatient Sample; HCUP: Healthcare Cost and Utilization Project; NEOMAT: Discharges with neonatal and/or maternal diagnoses and procedures.

We used ICD-9 code 571.8 (Table 1) to identify NAFLD population and excluded other causes of chronic liver disease such as alcoholic liver disease, autoimmune hepatitis, hemochromatosis, hepatitis B and C virus infection, primary biliary disease, toxic liver disease, and those with organ transplant (Figure 1). We used ICD-9 code 278.00, 278.01, 278.02, 278.03 to identify non-obese/overweight population (Table 1). We created two groups of NAFLD population based on BMI: 1. Obese group (defined as overweight or obese) and 2. Non-obese group (defined as normal or underweight). Our primary outcome was mortality among the non-obese/non-overweight hospitalized population which we will refer to in the text subsequently as “non-obese NAFLD”. Our secondary outcomes were liver cirrhosis and decompensated liver cirrhosis (hospitalizations with cirrhosis and any of hepatorenal syndrome, encephalopathy, ascites, variceal bleed, portal hypertension, and jaundice). We studied the prevalence and trends of hospitalizations in these groups of NAFLD patients. We also compared demographic and clinical characteristics to assess the differences between these two groups.

**Table 1:** ICD-9 codes for clinical conditions ICD: International Classification of Diseases; CM: Clinical Modification; NAFLD: Non-alcoholic fatty liver disease.

Clinical condition	ICD-9-CM code
NAFLD	571.8
Alcoholic liver disease	571.0
	571.1
	571.2
	571.3
Alcoholic use	303.x
	305.0x
Autoimmune hepatitis	571.42
Hemochromatosis	275.01
	275.02
	275.03
Hepatitis B virus	070.20
	070.21
	070.22
	070.23
	070.30
	070.31
	070.32
	070.33
Hepatitis C virus	070.41
	070.44
	070.51
	070.54
	070.70
	070.71
Previous organ donor	V59.6
	V59.9
Previous organ recipient	V42.7
	V42.9
Primary biliary cirrhosis	571.6
Toxic liver disease	571.41
	573.3
Cirrhosis	571.5
Ascites	789.51
	789.59
Variceal bleed	456.0
	456.2
Hepatorenal syndrome	572.4
Hepatic encephalopathy	572.2
Portal hypertension	572.3
Jaundice	782.4
Obesity/Overweight	278.00
	278.01
	278.02
	278.03

Statistical analysis

We used STATA version 15.0 (College Station, TX) for analysis. We reported the mean and standard deviation for continuous variables, and frequency and percentage for categorical variables. Statistical tests such as the chi-square test and Student’s t-test were used for comparisons across obesity groups. Trend analysis was derived using the Joinpoint regression analysis statistical software to derive annual percentage change (APC). The APC considers constant changes that occur over a specified period in which the rate of a disease change is seen in relation to the years assessed in percentages. The Joinpoint software takes trend data and based on the maximum number of Joinpoint supplied by the user, it (Joinpoint) fits the data into segments, enabling the users to assess if the apparent change in trend is statistically significant [10]. All analysis was performed with strata and weight to account for the complex clustered sampling methodology.

## Results

Baseline characteristics

We identified a total of 194,787 hospitalizations with NAFLD over the five-year period from January 1st, 2010 to December 31st, 2014, of which 119,048 (61.1%) were non-obese/non-overweight. The mean age of the overall NAFLD population among US hospitalization in the US was 55 years. The non-obese population was older than the obese NAFLD group (57.5 vs 51.5 years, p < 0.001) and a higher proportion of males (43.3% vs 36.1%, p < 0.0001) than obese NAFLD hospitalizations. Compared to the obese hospitalizations, the non-obese NAFLD hospitalizations were less likely to have co-morbidities including diabetes, hypertension, and dyslipidemia (Table 2). Non-obese NAFLD had a higher proportion of cirrhosis (8.4 vs 6.0%, p < 0.001), hepatorenal syndrome (0.7 vs 0.4%, p < 0.001), jaundice (1.5 vs 0.9%, p < 0.001), ascites (7.6 vs 3.9%, p < 0.001), hepatic encephalopathy (5.1 vs 3.2%, p < 0.0001), and portal hypertension (5.0 vs 3.4%, p < 0.001).

**Table 2:** Baseline characteristics of hospitalizations with NAFLD in the United States NAFLD: Non-alcoholic fatty liver disease; N: Weight average estimates; n: Sample number; %: Percentage.

Variable	Overall NAFLD (n = 194,787) (N = 953,598)	Non-Obese NAFLD	Obese NAFLD	P-Value
Age, years	55.2 (±0.1)	57.5 (±0.1)	51.5 (±0.1)	<0.0001
Sex, %				
Male	40.5	43.3	36.1	
Female	59.5	56.7	63.9	<0.0001
Race, %				
White	71.3	71.4	71.0	
Black	8.8	8.1	9.9	
Hispanic	14.3	14.2	14.4	
Other	19.9	20.5	19.1	<0.0001
Comorbidities, %				
Diabetes Mellitus	48.3	44.2	54.8	<0.0001
Hypertension	60.9	58.1	65.2	<0.0001
Dyslipidemia	40.3	37.8	44.2	<0.0001
Liver decompensation, %				
Cirrhosis	7.4	8.4	6.0	<0.0001
Hepatorenal syndrome	0.6	0.7	0.4	<0.0001
Encephalopathy	4.4	5.1	3.2	<0.0001
Portal hypertension	4.3	5.0	3.4	<0.0001
Jaundice	1.3	1.5	0.9	<0.0001
Ascites	6.2	7.6	3.9	<0.0001
Variceal bleed	0.2	0.2	0.1	<0.0001
Income, %				
First quartile	27.4	27.7	27.0	
Second quartile	26.1	25.9	26.3	
Third quartile	25.1	24.7	25.7	
Fourth quartile	21.4	21.6	21.0	<0.0001
Insurance, %				
Government	51.2	55.0	45.2	
Private	38.5	34.5	44.6	
Self-pay	6.5	6.5	6.4	
Others	3.9	3.9	3.8	<0.0001
Region, %				
Northeast	15.8	16.7	14.4	
Mid-West/North Central	21.2	20.8	21.7	
South	41.3	41.7	40.7	
West	21.7	20.8	23.2	<0.0001
Hospital Teaching Status, %				
Rural	9.4	10.1	8.4	
Urban non-teaching	39.2	38.7	40.1	
Urban teaching	51.4	51.3	52.5	<0.0001
Discharge disposition, %				
Home/Home health	88.4	86.9	90.8	
Others	11.6	13.1	9.2	<0.0001
Length of stay, days	4.8 (±0.02)	5.1 (±0.03)	4.4 (±0.04)	<0.0001
Mortality, %	1.4	1.8	0.8	<0.0001
Cost	13,074.0 (±133.8)	12,980.8 (151.1)	13,221.1 (±139.9)	0.9992

Outcomes of non-obese NAFLD hospitalizations

Multivariate logistic regression showed higher odds of mortality (OR 1.49, p < 0.001) among the non-obese NAFLD after adjusting for age, sex, race, co-morbidities, liver cirrhosis/decompensation) (Table 3). It also revealed that non-obese hospitalizations had higher odds of cirrhosis (OR 1.30, p < 0.001) and decompensated cirrhosis (OR 1.30, p < 0.001) after adjusting for age, sex, race, diabetes mellitus, and dyslipidemia. Increasing age remained an independent predictor of mortality among the NAFLD population irrespective of their weight classification. Those with diabetes mellitus were also more likely to have decompensated liver cirrhosis (OR 2.60, p < 0.001).

**Table 3:** Factors associated with mortality in non-obese NAFLD patients NAFLD: Non-alcoholic fatty liver disease; OR: Odds ratio; CI: Confidence interval; %: Percentage.

Variable	Crude OR (95% CI)	P-Value	Adjusted OR (95% CI)	P-Value
Obese NAFLD	Reference		Reference	
No-obese NAFLD	2.13 (1.94-2.35)	<0.001	1.49 (1.35-1.65)	<0.001
Age (years)				
<40	Reference			
40-65	2.17 (1.84-2.58)	<0.001	2.13 (1.79-2.54)	<0.001
≥65	5.27 (4.47-6.22)	<0.001	4.67 (3.90-5.59)	<0.001
Sex				
Male	Reference			
Female	0.93 (0.86-1.01)	0.067	0.89 (0.82-0.97)	0.005
Race				
White	Reference			
Black	0.83 (0.72-0.96)	0.012	1.20 (1.04-1.40)	0.014
Other	0.83 (0.75-0.93)	0.001	0.96 (0.87-1.07)	0.495
Comorbidities				
Diabetes Mellitus	0.88 (0.82-0.96)	0.002	0.83 (0.76-0.91)	<0.001
Hypertension	0.79 (0.73-0.86)	<0.001	0.77 (0.70-0.84)	<0.001
Dyslipidemia	0.57 (0.53-0.62)	<0.001	0.64 (0.59-0.70)	<0.001
Cirrhosis	3.36 (3.05-3.71)	<0.001	1.05 (0.92-1.21)	0.463
Liver decompensation				
Hepatorenal syndrome	20.11 (17.30-23.27)	<0.001	5.42 (4.45-6.60)	<0.001
Encephalopathy	5.94 (5.37-6.55)	<0.001	2.32 (2.00-2.69)	<0.001
Portal hypertension	3.21 (2.85-3.61)	<0.001	0.91 (0.78-1.05)	0.219
Jaundice	3.68 (3.04-4.46)	<0.001	2.48 (1.99-3.09)	<0.001
Ascites	5.56 (5.10-6.06)	<0.001	2.49 (2.18-2.85)	<0.001
Variceal bleed	3.24 (1.87-5.63)	<0.001	1.87 (1.03-3.42)	<0.001

Trends in non-obese NAFLD hospitalizations

**Figure 2:**
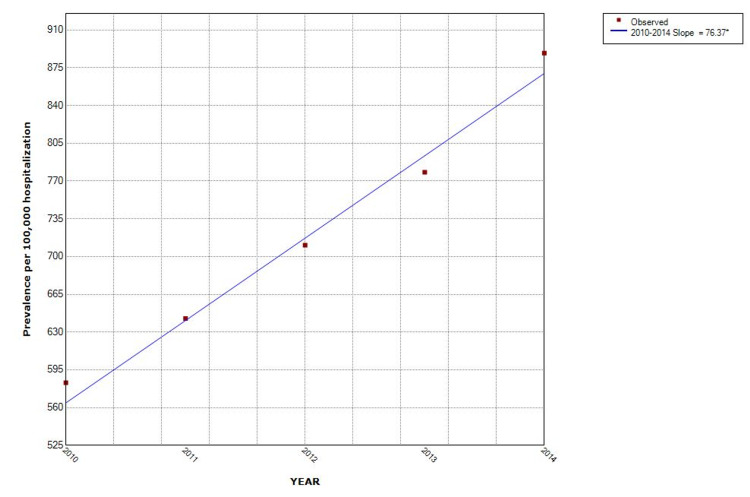
Trends in NAFLD hospitalizations in the US from 2010 to 2014 NAFLD: Non-Alcoholic Fatty Liver Disease

**Figure 3:**
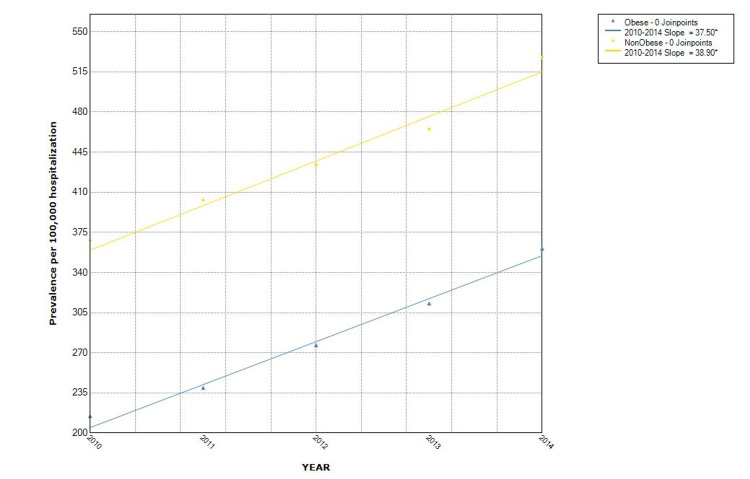
Trends in hospitalizations with NAFLD by obesity group in the US from 2010 to 2014 NAFLD: Non-Alcoholic Fatty Liver Disease

**Figure 4:**
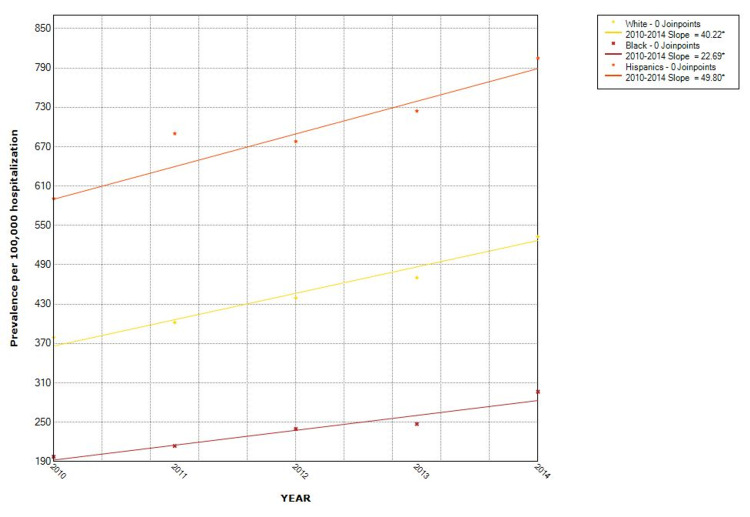
Trends in hospitalizations with NAFLD in non-obese by race in the US from 2010 to 2014 NAFLD: Non-Alcoholic Fatty Liver Disease

**Figure 5:**
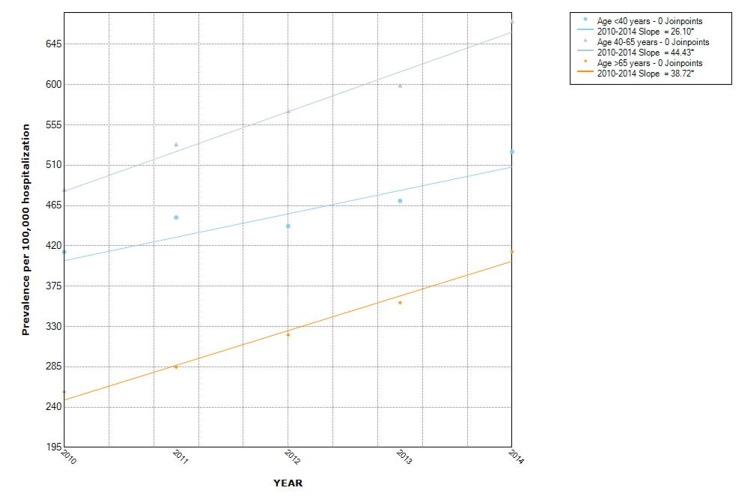
Trends in hospitalizations with NAFLD in non-obese by age in the US from 2010 to 2014 NAFLD: Non-Alcoholic Fatty Liver Disease

**Figure 6:**
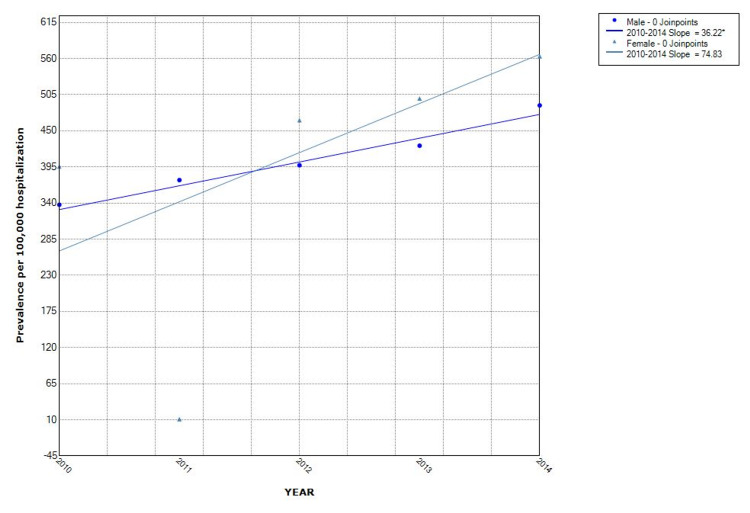
Trends in hospitalizations with NAFLD in non-obese by gender in the US from 2010 to 2014 NAFLD: Non-Alcoholic Fatty Liver Disease

The proportion of hospitalizations with NAFLD as well as non-obese NAFLD was observed to increase from year to year. Over the five-study period, the proportion of NAFLD in the non-obese population increased by 43% (from 368/100,000 in 2010 to 527/100,000 in 2014). The Joinpoint analysis revealed the annual percentage change (APC) of 76.4 (p = 0.001) in all NAFLD hospitalizations, with APC of 38.9 (p = 0.003) in the non-obese group and 37.5 (p = 0.001) in the obese category (Figures 2, 3). Hispanics had the highest proportion while Blacks had the lowest proportion when stratified by race. Hispanic also had the highest APC (Figure 4). The Joinpoint analysis showed higher APC in 40-65 and >65 years old, showing that these groups' rising trend is higher (Figure 5). It was interesting to see that males had a higher prevalence of NAFLD hospitalizations prior to 2012 while that of females became higher from the year 2012 but the overall APC was much higher in the females (female APC 74.8, p = 0.252; male APC 36.2, p = 0.004, Figure 6).

## Discussion

This study showed an increase in the proportion of hospitalizations with an associated diagnosis of NAFLD over the five-year study period from 2010 to 2014. This proportion is rising in both the non-obese and the obese patient populations. The annual percentage change was 38.9 (p = 0.0027) in the non-obese population. The rising proportion of hospitalizations with an associated diagnosis of NAFLD is similar to that from other studies but this is the first large data that looked at the trend of NAFLD in the non-obese/non-overweight patients. The odds of NAFLD in non-obese/overweight patients is higher with increasing age, but they are less likely to have hypertension, diabetes mellitus, and hyperlipidemia when compared to the obese/overweight population. Although a majority of prior studies had similar findings especially with regards to metabolic profile [11-14], some studies showed no difference in the mean age or sex distribution of NAFLD in both groups [13].

Multivariate logistic regression showed higher odds of mortality (OR 1.49, p < 0.001) among the non-obese NAFLD after adjusting for age, sex, race, co-morbidities, liver cirrhosis/decompensation. The higher odds of mortality may be related to increased waist circumference but unfortunately, our database did not have information on waist circumference. A study done by Golabi et al. showed that NAFLD in lean individuals had higher odds of all-cause mortality. However, these patients were also obese by waist circumference [15].

Our study also showed a higher odds of liver cirrhosis (OR 1.30, p < 0.001) among non-obese/non-overweight patients with NAFLD than those who were obese/overweight after adjusting for age, sex, and other co-morbidities. Other studies have revealed that non-obese NAFLD patients were less likely to have advanced fibrosis or cirrhosis [12, 16]. However, another study that looked at liver histology reported lean patients had higher rates of cirrhosis [13]. This suggests that we are yet to fully understand the progression of the disease. Also, important to note is that our study did not look at non-hospitalized patients with NAFLD.

The trend in rising prevalence as observed in our study is similar to that seen in other studies. It is unclear if this is due to increasing prevalence or just increased awareness leading to physicians paying more attention to making a diagnosis of NAFLD. In our study, the prevalence was higher among Hispanics and lowest among Blacks, which corroborates the findings of some of the other studies [17, 18]. This may show that the driver of NAFLD may be more than metabolic syndrome as the prevalence of hypertension and obesity is higher in the Black population [17, 19, 20]. The pattern seen with age was also noticed in the prevalence of NAFLD in the general population with the highest incidence in the 45-64-years group [21].

The limitations of this study include those associated with cross-sectional studies. We also did not have laboratory data or actual weight and heights to calculate BMIs and there may have been errors in coding the diagnosis. Also, important to note is that obese patients are more likely to have other metabolic and cardiovascular complications for which they may be hospitalized leading to a spurious observation that they were less likely to be hospitalized for cirrhotic complications. However, a large sample size and a database with demographic data across the United States is a strength of this study.

## Conclusions

There is a continued rise in the prevalence of non-obese NAFLD hospitalizations in the US, particularly amongst females. Non-obese NAFLD hospitalizations were older, less likely to have hypertension and diabetes, but more commonly had decompensated liver disease including jaundice, ascites, variceal bleed, and hepatic encephalopathy compared to obese NAFLD hospitalizations. Further prospective studies are needed to better characterize these patients to enable early detection, treatment, and reduction in complications of liver disease.
